# Adaptation of unified protocol treatment for transdiagnostic disorders in Pakistan: A heuristic framework

**DOI:** 10.1371/journal.pone.0308981

**Published:** 2024-09-30

**Authors:** Asma Nisa, Salma Siddiqui, Amantia A. Ametaj, Fahad Khan

**Affiliations:** 1 Department of Behavioral Sciences, School of Social Sciences and Humanities, National University of Sciences and Technology, Islamabad, Pakistan; 2 Institute for Health Equity and Social Justice, Northeastern University, Boston, Massachusetts, United States of America; 3 Khalil Centre, Lombard, Illinois, United States of America; University Hospital Marques de Valdecilla, SPAIN

## Abstract

The access to evidence-based treatments for mental health problems is limited in low-resource settings. Transdiagnostic approaches, such as the Unified Protocol (UP), are a potential solution for these settings because they are multi-problem focused, modular, flexible, and have low complexity. This study aimed to adapt UP to the mental health context of an urban speciality clinic in Pakistan using a four-step process of heuristic framework. The study employed an iterative and stakeholder-based approach to align the protocol with local values, language, and needs. Primarily, the proposed modifications focus on language use, matching literacy level, graphical illustrations, and relevance of examples. A multi-method approach including expert review, cognitive interviewing, and adaptation testing ensured cultural equivalence. Participants diagnosed with depression and anxiety were provided culturally adapted treatment (N = 15) at the testing phase. Findings indicated that the participants not only experienced significant reductions in symptoms of depression and anxiety but also found the culturally adapted UP to be easy to understand, culturally relevant, and engaging. This study provides evidence that the UP can be culturally adapted and used in the mental health context of Pakistan. The findings suggest that the UP is a promising intervention for individuals with depression and anxiety in low-resource settings.

## Introduction

Mental health disorders are a major global concern, affecting one in eight people worldwide [1]. Depression and anxiety are the most common, impacting over 280 million people [2]. These disorders often co-occur, sharing vulnerabilities such as negative affectivity [3], which can lead to severe and prolonged clinical trajectory, heightened disability, and elevated suicide risk [4, 5]. This underscores the need for improved mental health treatments.

Cognitive behavior therapy (CBT) presents a disorder-specific, manualized intervention, demonstrated to be efficacious for both anxiety and depressive disorders [6, 7]. However, comorbidity or heterogeneity within diagnostic categories often create challenges for adapting and ordering different CBT manuals [8]. The challenge of dissemination and implementation is significantly amplified since training therapists in multiple disorder-specific protocols is costly and time-consuming, especially in resource-scarce settings [9]. This highlights the need for refined treatment modalities.

The recently emerged transdiagnostic approach suggests an efficient method for addressing and managing the co-occurrence of multiple disorders [10, 11]. This approach is based on the premise that common underlying mechanisms lead to the manifestation and maintenance of symptoms across different psychological disorders [12]. Transdiagnostic practices offer an explanation for complex causal models for common psychiatric disorders, including but not limited to mood, anxiety, and substance use disorders [13, 14], and have been shown to be effective for a variety of mental disorders [12, 15–17].

The Unified Protocol (UP) is a type of transdiagnostic CBT approach that targets the core vulnerabilities underlying anxiety and depression, and related emotional disorders [18, 19]. The Diagnostic and Statistical Manual of Mental Disorders, Fifth Edition (DSM-5) classify emotional disorders as a broad category marked by emotion regulation difficulties such as anxiety and mood disorders (major depressive disorder, persistent depression, agoraphobia, panic disorder, social anxiety disorder, generalized anxiety disorder, specific phobias and related conditions like post-traumatic stress disorder (PTSD) and obsessive-compulsive disorder (OCD) [20]. These disorders often involve maladaptive coping strategies to control emotions that can exacerbate the disorder’s symptoms [18]. UP interventions focus on maladaptive emotions and dysfunctional emotion regulation strategies, aiming to equip individuals with adaptive skills for understanding and responding to their emotional experiences [21]. These skills include promoting flexibility in emotional responses, encouraging awareness and acceptance of emotions, and identifying and modifying unhelpful thoughts and behaviors [22].

The UP treatment for emotional disorders contains 8 distinct modules, typically delivered in 12–16 sessions [23]. The treatment begins with two complimentary modules dedicated to psychoeducation and goal setting. Following this, five core treatment modules are introduced, including mindful emotion awareness, cognitive flexibility, countering emotional behaviors, awareness and tolerance of physical sensations, and emotion exposures. An optional relapse prevention module is also available, providing individuals with tools to sustain the progress made during treatment.

A growing body of evidence supports UP’s efficacy across varied clinical settings and disorders. Studies have found that individuals treated with UP exhibited significant improvements in their emotion regulation capacities and a decrease in their symptoms [21, 23, 24]. UP has also been found to be effective not only in Western countries but other diverse cultures, such as Japan, Spain, Brazil, and Iran [25–28]. These various cultural contexts underscore UP’s potential in addressing mental health concerns worldwide, indicating the necessity for its ongoing adaptation and application.

### Cultural adaptation of evidence-based interventions in Pakistan

Culture impacts beliefs, understandings, and responses towards mental health concerns, as well as the propensity to seek help and response to treatment [29]; therefore, culturally adapted interventions resonate with the lived experiences of the target population, enhancing effectiveness, treatment adherence, and outcomes [30, 31]. Pakistan is a low-resource country with a high burden of mental illness, where the government’s mental health expenditure is just 0.4% of the Gross Domestic Product [32]. Like many low-income countries, Pakistan has limited access to evidence-based mental health programs [33]. The limited resources and professionals available make it challenging to address mental health disorders with conventional individual therapies [34].

The UP has the potential to be an effective treatment for Pakistanis due to several reasons. First, depression, anxiety, and PTSD are the most common mental health disorders in Pakistan [35], which the UP is efficacious at treating [21, 36]. Second, the UP can be flexible and adaptable to diverse cultural contexts [37]. In addition, the UP teaches individuals how to identify, understand, manage and tolerate their emotions, which is aligned with Pakistani cultural norms and values of emotional regulation [38]. Fourth, it is an efficient and cost-effective option for a broad range of emotional disorders, which would be beneficial for limited available resources for mental health available in Pakistan [9], including the dearth of trained therapists in evidence-based interventions [34].

Considering the UP demonstrated effectiveness, flexibility, and adaptability to diverse cultural contexts, this study aimed to adapt the UP for the Pakistani setting. The Heuristic Framework for Cultural Adaptation, as outlined by Barrera and colleagues (2013), presents a structured approach for adapting evidence-based treatments (EBTs) to make them more culturally suitable for specific groups [39]. It follows a four-stage process that begins with gathering data on the cultural background of the target population, including their norms, values, beliefs, and practices. This initial stage helps identify their particular needs and preferences. Second stage encompasses the cultural adaptation of the intervention by integrating relevant cultural elements to create an initially culturally appropriate version. The third stage is a pilot testing phase where the adapted intervention is assessed with a small sample from the target population. During this stage, feedback is collected to evaluate the overall cultural appropriateness of the intervention. Finally, based on this feedback, further refinement is undertaken to ensure an optimal alignment with the target culture in order to enhance its effectiveness over time. This framework allows for the integration of various dimensions in an operationally and systematically effective way [40]. The principles of this method align with the overarching stages of cultural adaptation recognized across a broad spectrum of public health studies [40, 41].

To enhance the treatment’s impact and acceptability among the target beneficiaries, this study sought to identify necessary modifications to the UP. Stakeholder engagement was prioritized, including a UP developer, mental health practitioners, native community experts, patients, and trial participants. Stakeholders contributed as equal partners via community-based participatory research [42]. The authors describe the modifications and decision-making process, which relied on consensus building, stakeholder feedback, and empirical research.

## Method and results

The method section will provide a detailed explanation of a four-stage heuristic framework procedure for modifying the UP Treatment. Each stage within this framework will have its own distinct sample, instruments, procedure, data analysis approach, and results. Therefore, the methods section will present details on each stage individually before proceeding to the next one. This will be followed by an integrated discussion section that evaluates the findings from all four stages collectively.

### Stage 1: Information gathering

The initial step involved collecting relevant data and knowledge to understand the context and needs of the treatment in the region. This was followed by the identification of the suitable treatment approach and the potential cultural variances and discrepancies in the protocol to make it more culturally appropriate for the targeted population.

#### Sample

*Adaptation committee*. This committee consisted of key stakeholders including mental health professionals, community experts, language expert, UP expert and research method expert (see Table 1 for details). In order to preserve the core components and essence of the treatment, this committee thoroughly examined all the stages of the adaptation. It also aimed to scrutinize procedural methods in a manner that ensures impartiality and openness. The committee operated through monthly meetings.

**Table 1:** Demographics of adaptation committee members, local resources and bilingual translators.

Members	Gender	Education	Relevant Experience
**Mental Health Professionals (n = 4)**	2 Males, 2 Females	MS in Clinical Psychology (2), PhD in Psychology (2)	Expertise in culturally adapting, translating, and establishing the reliability and validating mental health interventions for Pakistani contexts. Area of Specialization (depression, anxiety, trauma, somatoform, mood disorders); Prior experience with CBT (all Yes)
**Community Experts (n = 2)**	2 Females	Masters in Psychology	9 years training mental health first-aid providers; Area of community work (Rural: adults from low- and middle-income backgrounds with low literacy rates)
**Language Expert (n = 1)**	1 Female	Masters in Linguistics	Prior experience translating mental health or healthcare materials; Proven track record of delivering high-quality translations; Fluency in both source and target languages; Understanding of the target audience’s cultural context.
**UP Expert** **(n = 1)**	Female	PhD in Clinical Psychology	Global mental health research, improving detection and access to treatment for common mental health disorders (traumatic stress, anxiety, depression) in low-resource settings; Member of the transdiagnostic treatment lab; Unified Treatment Protocol expert; Intervention adaptation and implementation in different cultural contexts
**Research Method Expert (n = 1)**	Male	PhD in Psychology	Expertise in combining quantitative and qualitative data collection methods; Knowledge of mental health research ethics, data collection methods for sensitive topics, and working with vulnerable populations.
**Psychologists (n = 5)**	4 Females, 1 Male	MS Clinical psychology (2), MS Psychology (1), PhD Psychology (2)	Experience working with adults with psychological problems; Experience working with diverse populations and understanding cultural influences on mental health; Experience in specific therapeutic approach (e.g., CBT, Dialectical Behaviour Therapy). Knowledge of psychological assessment to evaluate protocol tools.
**Community Experts (n = 3)**	2 Females, 1 Males	Master of Public Health (2), Masters in Psychology (1)	Community work (rural, low-income, low literacy); Knowledge about the community’s demographics, cultural values, mental health needs, resources, and challenges (age, income, education levels); Adapted interventions to be culturally appropriate and recognizing the specific mental health needs of the community.
**Patients (n = 3)**	1 Male, 2 Females	Intermediate Degree (1), Bachelor’s Degrees (2)	Treatment duration (psychotherapy): Male patient: 8 months; Female patients: 2 years and 3 years, respectively. Number of therapy sessions: 23, 80, and >100 sessions respectively; Prior diagnoses: Generalized Anxiety Disorder (GAD) (n = 2), Major Depressive Disorder (MDD) (Recurrent) (n = 1)
**Bilingual Translators** **(n = 3)**	2 Females, 1 Male	MS Clinical Psychology	Bilingual and native Urdu speakers, Expertise in culturally adapting, translating, and establishing the reliability and validating mental health interventions for Pakistani contexts, Publications in Urdu newspapers

*Local sources*. Local sources included psychologists (n = 5), community professionals (n = 3) with more than 10 years of experience to examine the content of the UP and to determine the need for translation and/or adaptation of the treatment. This also included patients (n = 3) who had successfully undergone psychotherapy for depression and/or anxiety to provide insights into their lived experiences.

*Literature review*. A literature review of relevant materials was conducted, this included academic literature retrieved from databases and non-academic sources such as news articles, policy documents, and reports from pertinent Pakistani organizations and government agencies to ensure a well-rounded understanding of the target population’s challenges and circumstances.

#### Instruments

*Semi-structured interview schedule*. The researchers developed a semi-structured interview guide through a systematic approach. Initially, the primary researcher identified relevant interview topics for different stakeholder groups (mental health professionals, community experts, and patients) in line with the research goals by conducting a thorough review of existing literature. The draft interview guide was then reviewed by a mental health expert specialized in global mental health (third author), whose expertise matched well with the study’s focus on enhancing access to treatments for common mental health disorders in low-resource settings, ensuring the interview questions were appropriate for this context. Finally, a qualitative research expert evaluated the interview schedule to ensure its clarity, flow, and effectiveness in capturing participant perspectives.

The semi-structured interviews, conducted at different stages (see Table 2 for details), collected information from various stakeholders on various topics. From mental health professionals the interview explored prevalent mental health disorders, the effectiveness of CBT compared to other treatments, cultural influences on symptom presentation, necessary treatment modifications, applicability of the UP in our context. Community professionals were asked about the community perceptions of mental health, stigma surrounding disorders and treatments, current treatment implementation and adaptation, challenges in diagnosis and treatment, and recommendations for adapting the UP. Patients were inquired about the interpretations of their illness, symbolic meanings attached to their condition, past treatment experiences, and treatment preferences for a culturally-adapted approach. The complete interview guide with all key stakeholders and sources can be found in S1 Appendix.

**Table 2:** Interview timing by each adaptation stage and stakeholder.

Stage	Stakeholders Interviewed	Type of Interview	Purpose
**Stage 1: Information gathering**	Mental health professionals, Community experts, Patients	Semi-structured (qualitative)	Needs assessment: Identify prevalent diagnoses and emotional experiences to guide treatment selection
	Local Sources	Semi-structured (qualitative)	Identify cultural discrepancies or aspects requiring adaptation to better suit the target population’s needs
**Stage 2: Preliminary adaptation design**	Mental health professionals, Community experts, Patients	Likert scale (quantitative) & 1 Open-ended question for additional feedback (qualitative)	Assess cultural appropriateness and potential need for further adaptation of the UP protocol
**Stage 3 and 4: Preliminary adaptation testing and refinement**	Patients (N = 15)	Likert scale (quantitative) & Semi-structured interviews (qualitative)	Evaluate participant experience with the culturally adapted UP modules. Assess clarity, cultural relevance, effectiveness of exercises, and overall program perception

#### Procedure

To understand the potential needs and context of the targeted population, relevant literature review and interviews with the adaptation committee and patients were conducted. Each interview lasted approximately one hour, was recorded, and transcribed by an independent researcher.

The process of obtaining information was essential in determining which intervention was best for the target audience. This process informed the decision to select the UP-treatment protocol [23], as it best addressed the specific mental health needs identified.

Following this, a thorough review took place with local sources, such as psychologists, community intervention experts and patients. These sources spent two weeks reviewing the protocol and identifying any cultural aspects that needed modification to better meet the needs of the target population. Following this, eight two-hour meetings were conducted to discuss their detailed feedback on various aspects of the protocol like understanding, cultural relevance, language use, core concepts, and accompanying materials. The primary researcher documented all feedback given during these meetings.

After reviewing all the gathered input, including local sources’ comments captured in meeting records, and conducting a thorough examination of current research, the adaptation committee made decisions on revising the protocol.

#### Data analysis

For analyzing recorded communication, content analysis method was employed to analyze the qualitative data gathered from the interviews.

#### Results

Insights from mental health practitioners and community specialists highlighted the underutilization of EBTs for psychological disorders in routine care settings. They expressed their observations regarding a high occurrence of disorders such as depression, anxiety, PTSD, and somatic disorders among their patients, often exhibiting high comorbidity. They further shared their concerns about the difficulties in managing complex treatment plans due to a high patient-doctor ratio, which can notably affect a patient’s treatment compliance. Moreover, they emphasized the necessity for EBTs that can effectively manage various mental health conditions.

Concerns were raised by patients (n = 3) regarding the high costs, and time-consuming nature of the therapy, along with the challenge of regular therapist visits due to the societal stigma connected to mental health disorders. Hence, they suggested that the proposed solution needs to be a treatment with a limited number of sessions and is sensitive to cultural nuances.

The insights gathered from community professionals suggest that most clients attending care at health centres speak primarily in Urdu. Literature reviews also highlighted that most of these patients do not comprehend English terminology, leading to hurdles in delivering quality care [43]. Moreover, our literature review indicated key factors to consider during the adaptation of interventions in mental health care, particularly tailoring approaches to match the clients’ education and literacy levels. Specifically, considerations should include unfamiliarity with Western psychological concepts, limited English skills, minimal education, and culturally specific expressions of distress when undertaking the adaptation process [44].

Specific modifications that were identified for the UP-treatment protocol involved several aspects:

*Terminology review*. Recognizing potential challenges in directly translating some UP concepts into Urdu, the team consulted relevant research papers and engaged in discussions with Pakistani mental health professionals. This aimed to identify culturally appropriate terminology. For instance, "non-judgmental awareness" was translated as "being aware of something without labelling it as good or bad." Additionally, they identified expressions in Urdu that capture the concept of “subtle behavioral avoidance”, as to "avoiding something in a subdued manner." This phrase captures the essence of acting on avoidance urges without drawing attention.

The concept of “anchoring in the present moment” when translated in literal terms becomes closer to "living in the present" which doesn’t capture the essence of the concept of being firmly placed or grounded in the current moment. Hence the team suggested a conceptual translation that emphasizes “not just existing in the now, but also being secure and focused within it.”

*Example adaptation*. The team reviewed the original UP examples and case studies, identifying those potentially irrelevant to the Pakistani context. Alternative examples were then suggested based on research on common cultural references and situations in Pakistan. Firstly, to enhance cultural relevance the team suggested that the names in the examples could be changed to local names. Secondly, for social anxiety, examples related to dating could be replaced with scenarios more relevant to Pakistani culture, such as attending social events or family gatherings. Dating in Muslim communities often carries a stigma and cultural norms may forbid it [45].

Additionally, the World Health Organization reports that the average adult in Pakistan consumes roughly 0.2 Liters of pure alcohol per year, which is significantly lower than the global average of 6.4 Liters per year [46]. Due to the lower prevalence of alcohol consumption in Pakistan, the references to alcohol were recommended to change to include a wider range of substances.

Furthermore, the team identified a gender imbalance in the original UP illustrations, where women are shown as working professionals. According to data from the UN’s National Report on the Status of Women in Pakistan, 2023, the female labour force participation rate in Pakistan is notably lower than the global average, standing at only 21% compared to the global average of 39% [47]. Given that a significant number of Pakistani women are homemakers, mental health experts proposed adjusting some examples to better reflect this demographic. For example, a scenario originally depicting a working woman experiencing obsessive compulsive symptoms could be modified to portray a homemaker encountering similar challenges to ensure that the examples connect with various demographic characteristics within our target population.

*Audio exercises*. The team further suggested to provide alternative audio and video options in for emotional awareness, acknowledging that not all individuals may appreciate music-based exercises. This approach caters to different preferences and guarantees access to effective exercises for enhancing emotional awareness. Therefore, they recommend in the workbook to include activities utilizing music, videos, movie clips, natural sounds, or a combination according to personal choice.

*Image selection*. Recognizing the potential for varying literacy levels within the target population, culturally appropriate illustrations were recommended to enhance understanding of core UP concepts. These illustrations could explain concepts like the three components of emotions (physiological, cognitive, and behavioral), and motivation enhancement to attend this treatment program and bringing change in their lives.

### Stage 2: Preliminary adaptation design

#### Sample

A team of experts in psychology and linguistics carried out the translation process. This included two bilingual translators and the lead researcher, who had strong qualifications in linguistics. Their previous work on translation projects was featured in Urdu newspapers, demonstrating their expertise. Additionally, their background in psychology helped them grasp the subtle details of the intervention more comprehensively.

#### Instruments

Stakeholders rated the translated-UP workbook on three indicators on a 1–5 Likert scale. This feedback focused on comprehension, cultural appropriateness, and the treatment materials’ overall usefulness for the target population. The sample question include: *How easy is it to understand the language used in the workbook*? *(1 = Very difficult*, *5 = Very easy)*. The complete scale can be found in S1 Appendix.

#### Procedure

The UP workbook was translated into Urdu, following this, stakeholder feedback was obtained for further refinement. Mental health experts were given the modified workbook and rating scale to gather feedback on the adapted materials. Revisions were made based on this feedback to finalize the protocol.

#### Results

The preliminary adaptation of the UP involved making several changes to the UP workbook as shown in Table 3.

**Table 3:** Discrepancy between the original protocol and the characteristics of the target population, and preliminary adaptation of the protocol.

Issues Identified	Description	Preliminary Adaptation
**Translation of English phrases**	The UP uses some English phrases that do not have direct translations in Urdu such as “non-judgemental”, “Present-focused”, “subtle avoidance”.	Workbook: The terms are clearly explained in the text to help the reader understand their use.
	Psychiatric labels, terms, and phrasal expressions were identified as difficult and not commonly used.	Replaced by simple language and more culturally relevant terms and phrases.
**Understanding of concepts**	People with lower levels of formal education and familiarity with reading material might not understand the concepts in the English language.	Workbook: Translated in Urdu language for better understanding of the content.
**Cultural relevance of examples**	Examples were not relevant to our target population.	Workbook: Examples relevant to the cultural experiences and characteristics of the population were added.
**Visual aids**	Module 1: Making treatment motivation simpler for patients, considering the initial distress, and planning future actions.	Workbook: Chapter 4 ‐ Incorporated visual aids for easier understanding of motivation and goal setting.
	Module 2: Understanding emotions; Use visuals for better emotion comprehension.	Chapter 5: pictorial illustrations added to explain a three-component model for emotion regulation.
	Module 6: Understanding Physical Sensations; Images needed for exercises like push-ups, burpees, and squats.	Chapter 10: Pictures indicating how to perform each interoceptive exposure were added.
**Audio exercises**	Module 3: Emotional awareness; Some individuals may not find music appealing, other audio/video exercise alternatives are essential.	Workbook: Chapter 7: Exercises with music, videos or movie clips, and natural sounds were included in sessions and as homework based on personal preferences.

### Stage 3: Preliminary adaptation test

In this stage the UP protocol was tested and refined iteratively based on participants’ feedback to make final adjustments to the protocol.

#### Sample

The recruitment period for this phase commenced on December 15, 2022, and concluded on January 15, 2023. To recruit participants for the preliminary adaptation test from Islamabad and Rawalpindi, the study employed a purposive sampling method. Flyers were shared on social media platforms like WhatsApp groups, Facebook, and LinkedIn, aimed to reach a diverse range of people from the general population, such as students, professionals, and other community members. These flyers specified that the study sessions would be conducted in-person, ensuring only residents of these cities could participate.

Inclusion criteria were: (a) 18 years of age or older and (b) evidence of moderate to severe anxious and/or depressive symptomatology based on BDI-II [48] and BAI [49]. Exclusion criteria were: (a) individuals with suicidal risk, (b) comorbidity with a pervasive developmental disorder, psychotic disorder, or severe physical illness, and (c) individuals receiving concurrent psychotherapy or psychopharmacological treatments.

Based on the criteria, out of the 22 people initially recruited, 17 participants were retained while two with suicide risk were excluded with appropriate referrals. Two participants withdrew voluntarily resulting in a total of 15 participants in the final sample. Table 4 shows the demographic information of the participants.

**Table 4:** Demographic characteristics of the study participants.

Characteristic	(N = 15)
Age (M Years, SD)	27.53 (9.15)
Gender	
Female	11(74)
Male	4(26)
Marital status	
Married	6(40)
Unmarried	9(60)
Education	
Intermediate	4(26.67)
Bachelor’s	9(60)
Master’s or Higher	2(13.33)
Employment status	
Unemployed	8(53)
Employed	7(47)
Diagnosis based on DSM-5-TR	
Major Depressive Disorder (Total)	3(20)
Major Depressive Episode, Single Episode	1(6.7)
Major Depressive Episode, Recurrent Episode	2(13.3)
Anxiety Disorders (Total)	6(40)
Generalized Anxiety Disorder	3(20.0)
Panic Disorder	2(13.3)
Social Anxiety Disorder	1(6.7)
Comorbidity of depression and anxiety (Total)	6(40)
MDD with Anxious Distress Specifier	4(26.7)
Generalized Anxiety Disorder with MDD	2(13.3)

#### Instruments

*Treatment outcome measures*. *Demographic survey*: The survey’s demographic section collected data on a range of participant characteristics, such as age, gender, marital status, educational background and employment status.

*Beck anxiety inventory*: The BAI is a self-report tool that assesses anxiety symptom severity through a 21-item questionnaire, with higher scores indicating higher anxiety levels [48]. The Urdu version of BAI was translated by Raza and demonstrated high reliability in the current research α = 0.90 [50].

*Beck depression inventory-II*: BDI-II is a 21-item self-report questionnaire to evaluate depressive symptoms, with higher scores indicating severe symptoms [49]. The Urdu version utilized in this study, demonstrated high reliability α = 0.92 [51].

*Semi-structured clinical interview*: The study assessed participants for mental health conditions using the diagnostic criteria from the Diagnostic and Statistical Manual of Mental Disorders, Fifth Edition, Text Revision (DSM-5-TR) [52]. Participants were included if they met the criteria for Major Depressive Disorder and/or anxiety disorders, while those meeting the criteria for Substance Use Disorder, Bipolar Disorder, or Psychotic Disorders were excluded.

*Work and Social Adjustment Scale (WASA)*: It is a recognized measure of functional impairment, having 5 eight-point severity items, scoring up to 40 points in total severity [53]. This scale has wide validation across diverse psychiatric disorders. The Urdu version is also reliable with an accuracy score of α = 0.89.

*Difficulties in Emotion Regulation Scale (DERS)*: The DERS is a 36-item tool that contains 6 subscales. It measures a person’s competency in managing emotions and their emotional responses [54]. The subscales evaluate acceptance of emotional responses, purposeful behavior performance, impulse control, emotional awareness, emotion regulation strategies availability, and emotional clarity. The items score from 1 to 5, with higher values reflecting more severe emotion regulation issues. The Urdu version showed strong reliability in this study α = 0.87.

*Positive and Negative Affect Schedule (PANAS)*: This is a self-report measure that assesses the two broad dimensions of affect: positive affect and negative affect [55]. Designed as a 10-item scale, items are rated on a 5-point range, with higher scores indicating greater experience of the evaluated emotions. The current study used the Urdu version of this scale [56]. The positive and negative affect scales yield a reliability score of α = 0.84 and α = 0.86 for this study, respectively.

*Overall Depression Severity and Impairment Scale (ODSIS)*: It uses a 5-point Likert scale to evaluate the frequency, severity, and impacts of depression symptoms on pleasurable activities, work or school performance, and interpersonal relationships [57]. Scores range from 0–20, with higher scores indicating more severe depression and impairment. The Urdu version also indicated good reliability α = 0.89.

*Overall Anxiety Severity and Impairment Scale (OASIS)*: It is a 5-item scale, scored on a scale of 0–4 to assess the frequency and severity of symptoms, along with the impairments in enjoyable activities, work, school, and interpersonal relations [58]. The Urdu version yielded high reliability α = 0.90.

*UP workbook assessment*: *Likert scale & interviews*: Participants rated core concept clarity, workbook readability and terminology, homework, and cultural relevance of examples using a 5-point Likert scale (1 = disagree, 5 = agree). Additionally, semi-structured interviews explored areas like overall program perception, clarity of content, value of exercises, cultural connection of materials, participant engagement, difficulties experienced with the treatment protocol, and suggestions for improvement (for complete interview guide see S1 Appendix).

#### Procedure

*Adaptation testing procedure*. Participants for this research were recruited using a flyer that included a link to a Google Survey containing BDI-II [48] and BAI [49]. Upon completing the online survey, researchers screened the BDI-II and BAI scores to identify potential participants who met the initial criteria based on their self-reported symptoms. Eligible individuals were then contacted via email, which outlined the study’s objectives, eligibility confirmation, and included a link to schedule convenient time slot for a semi-structured clinical interview using the DSM-5-TR criteria for further diagnosis of depression and/or anxiety [52]. The interviews took place in the university’s therapy room.

Participants were provided with a detailed explanation of the study’s objectives and potential treatment before the interview. Informed consent was then obtained. This consent process included a written form completed before the questionnaire and verbal confirmation documented during the interview. The date and consent were recorded on a separate form with participant signatures. This approach to obtaining informed consent for the interview was approved by the ethics committee of the School of Social Sciences and Humanities (0988/Ethic/01/S3H/083/DBS).

They were also informed that treatment could start between 1 and 8 weeks. The participants were randomly assigned to one of three groups (n = 5 per group) using a random number generator to ensure an equal chance of being assigned to each group. The purpose of dividing the sample size of 15 participants into three groups of five each, was to facilitate an iterative approach to adaptation testing, allowing for ongoing refinement of the intervention based on participant feedback. Hence, the treatment was introduced in a staggered manner across three groups at different time points (four weeks apart).

*Participant feedback*. Participant feedback was collected after the end of every module (total of eight modules) on participants’ thoughts about the UP through mixed-methods, including a quantitative survey and a brief qualitative interview. Brief qualitative feedback was audio-recorded for consistency and transparency. The process was iterative in that qualitative and quantitative assessment findings from one group of participants were discussed with the adaptation committee and incorporated before administering the module to the next group of participants. This iterative approach to treatment adaptation was used to ensure that the treatment was tailored to the needs of the participants.

*Treatment*. The adapted-UP treatment was provided in Urdu, and participants attended 14 weekly in-person individual sessions in the university therapy room, each lasting 50–60 minutes. During these sessions, the therapist used a participatory inductive approach to simplify concepts, encouraged participants to relate their experiences to treatment concepts, and emphasized empathy, and validation. Monthly meetings with the program developer and study supervisor were conducted to discuss and modify the protocol to suit the patient’s needs and context.

*Therapist training*. The study therapist was a clinical psychologist (A. N.), who undertook an 18-week UP training and supervision from a protocol team expert (A. A. A.). Weekly sessions with the client were recorded, transcribed, translated, and reviewed by the expert to ensure adherence and efficacy.

#### Data analysis

For quantitative feedback, the mean was calculated from all three groups. The in-session qualitative feedback underwent a thorough process of content analysis by an independent expert. This involves extracting common themes pertinent to the workbook’s adaptation and categorizing them, to provide a better understanding of the overall process. Furthermore, peer review by another expert was employed to reduce bias. The preliminary treatment effects were assessed at three time points: pre-assessment (baseline), mid-assessment (7th week), and post-assessment (14th week) using a repeated measure ANOVA in SPSS 26 version.

### Stage 4: Adaptation refinement

**Fig 1:**
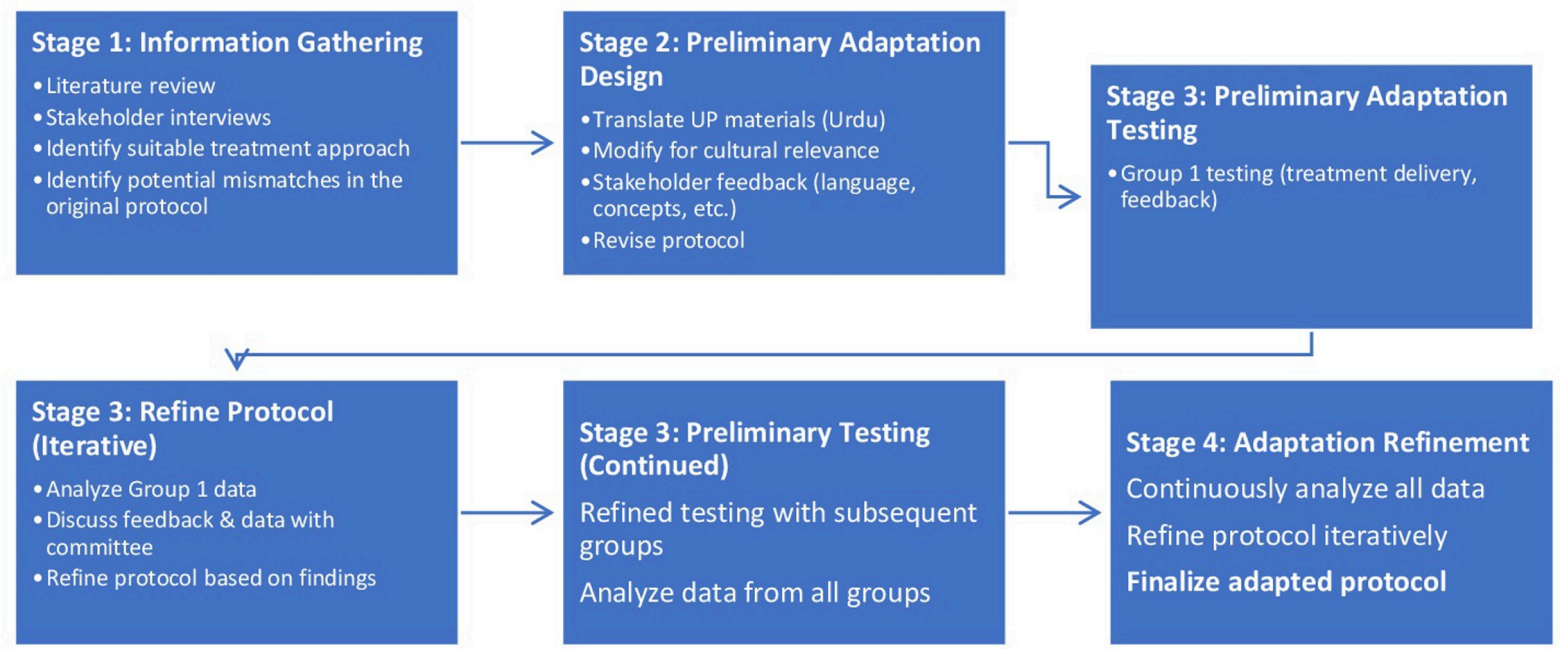
Multistage iterative cultural adaptation of the up intervention. This figure depicts a multistage iterative process for culturally adapting the UP intervention.

The researchers refined the adapted protocol iteratively, reflecting their belief that cultural adaptation is an ongoing process. This iterative refinement process involved continuous adjustments to the protocol based on feedback from the stakeholders and data collected from a sample group in stage 3 (Fig 1).

#### Results

The qualitative results on the adaptation of UP revealed four main themes, including both the effective and challenging components of the adapted workbook and sessions. See the summary of key findings with representative participant quotations in S2 Appendix.

*Comprehensibility of the UP*. Participants found the main concepts presented in each session and the corresponding workbook to be understandable (n = 10). They appreciated the clear and concise explanations of the concepts, as well as the use of everyday language. For instance, one participant reported, "For the most part, I found the material to be quite manageable." Another participant said, "I was pleasantly surprised at how easy it was to understand the psychological concepts, even in Urdu. The use of everyday language made it very accessible."

*Content*: Participants feedback on the UP materials shows that they are easily understandable and effective. Participants found the explanations of psychological concepts clear and well-structured, even for those unfamiliar with the terminology. One user mentioned, "I didn’t find many concepts particularly difficult. The explanations were clear, and real-life examples helped a lot.". Another participant also emphasized “how helpful the use of real-life examples was in illustrating concepts". Additionally, participants appreciated having definitions or examples to explain new psychological terms. A participant remarked, "There were some new vocabulary terms related to psychology, but their explanation was very helpful. Now I understand terms like ’thinking traps’ and ’emotional awareness’ much better!". In general, these positive responses indicate that the UP materials effectively convey complex psychological concepts in an easy-to-understand manner.

*Sentence structure*: Feedback from participants regarding the sentence structure of the UP materials emphasizes its readability and user-friendliness. The sentences are described as clear and concise, avoiding unnecessary complexity. According to one participant, "The sentences were straightforward and not overly complicated." Additionally, they found it engaging and easy-to-follow writing style. One participant remarked, "The writing was engaging and felt more like a helpful guide than a textbook." This suggests that the materials present the information in an accessible manner. Lastly, users admired how the presentation style, along with clear explanations, contributed to overall comprehension. One user expressed this by saying, "Despite dealing with complex topics, it was presented in a very understandable way." These responses indicate that the sentence structure and writing style increase user engagement and understanding within UP materials.

*Facilitative effect of prior discussion*. Participants reported that discussing the content of the UP during sessions before reading it helped them to understand it better (n = 9). This was because the discussion allowed them to ask questions and clarify any areas of confusion. As one participant said that “discussing the content before reading it helps me to understand it better”. Another participant said, “the discussion helps me to retain the information better.”

*Enhanced understanding*: Pre-reading conversations provided an opportunity for participants to ask questions and clarify any initial confusion about the concepts. One participant mentioned, "Talking about the content before reading it improves my comprehension. There were a few terms I didn’t know, but by discussing them beforehand, it became easier to understand when they appeared in the reading." Discussing important terms ahead of time may have helped prepare participants for encountering them in the reading materials. This could have enhanced their understanding of the concepts as they were presented. Another participant observed, "I found the pre-reading discussions very beneficial. They introduced some of the main ideas so that when I read through the workbook, it felt like I already had a basic understanding which made it easier to grasp." Overall, these comments highlight how prior discussions played a valuable role in addressing uncertainties and acquainting participants with key concepts of the treatment.

*Enhanced information retention*: Discussing the content prior to reading appeared to be resulted in a more engaging learning experience. One participant noted, "The discussions before reading really enhanced my understanding of the material. It was not just passive reading; we were actively discussing the concepts, which made them feel more tangible and memorable." Pre-reading discussions helped participants establish connections between different aspects of the UP material, potentially leading to a more cohesive mental framework and better retention of information. Another participant said, "I believe that our discussions allowed me to grasp how everything was interconnected. We explored various concepts and their relationships, making it easier for me to recall the information when I read independently." These comments demonstrate how participants attributed their improved retention of information to active and interrelated learning experiences fostered by pre-reading discussions.

*Cultural relevance of the UP*. Participants reported that the examples and exercises given in the UP were culturally relevant (n = 10). They appreciated that the materials reflected their cultural context. This made the UP more engaging and relatable for the participants, as a participant highlighted that “the examples are relatable to my own experiences”, and “the materials reflect my cultural context.”

*Relatable examples*: Participants found the examples in the UP to be relevant to their own experiences and cultural background, leading to increased engagement and a sense of connection with the material. One participant expressed this by saying, "The examples in the workbook were excellent. They didn’t feel like generic situations; they felt like something that could really occur within our culture. It made it much simpler to grasp the concepts."

*Culturally appropriate*: The use of culturally appropriate examples was also valued by participants. This attention to cultural context likely enhanced the learning experience positively and meaningfully. A participant reported, "I felt that the materials’ examples truly took into account our cultural heritage. It wasn’t as if they were attempting to enforce foreign ideas on us. It felt pertinent and respectful." These statements show how incorporating culturally relevant examples and sensitivity contributed to a more engaging and meaningful learning experience for participants using UP materials.

*Assessment and practice*: *Quizzes and homework*. Feedback from the chapter quizzes revealed insights into their perceived effectiveness. Participants noted their value in reinforcing learning and assessing comprehension. One participant mentioned, "I found the end-of-chapter quizzes to be very beneficial. They prompted me to review the material I had just covered, serving as a helpful tool for evaluating my understanding." Similarly, feedback on homework assignments emphasized their usefulness in applying learned concepts to real-world scenarios. Participants viewed them as a practical method for connecting theory with practice. A user stated, "The weekly homework assignments offered an excellent opportunity to apply what I had been learning. They allowed me to see how the concepts could be utilized in real-life situations."

*Challenges with homework completion*: While some participants found the homework assignments valuable, some participants (n = 8) reported occasional difficulties in completing them. Participants with busy schedules faced difficulties in setting aside dedicated time for the homework, as expressed by a participant who stated, "Finding time to do the homework tasks amidst work and family responsibilities was challenging." Some participants found it tough to stay motivated to complete the homework when immediate results were not apparent. This lack of motivation could hinder consistent involvement. One participant mentioned, "At times, I struggle with being motivated to finish the homework assignments, especially if I don’t notice immediate changes. It’s difficult to keep focused on long-term advantages."

Table 5 further summarizes the specific feedback from three groups (Groups 1–3) on the UP materials and the corresponding iterative improvements made to enhance comprehensibility and suitability of the protocol.

**Table 5:** Iterative refinement of the UP through group-specific feedback.

Group 1 Feedback	Group 2 Feedback	Group 3 Feedback
**Identified difficult Urdu terminologies** (n = 6), which were replaced with simpler alternatives	**Identified difficult Urdu terminologies** (n = 2), which were replaced with simpler terminologies	No further alterations were made to the adapted material for Group 3. This suggests that the provided resources, which included simpler Urdu terminologies, were satisfactory in terms of comprehensibility and suitability for this group.
**Difficulties completing homework forms.** Filled out homework forms during the session to provide participants with instruction on how to complete them at home. Completed the first one or two columns of homework sheets in session for all subsequent participants to enhance their understanding of how to do the homework.	**Difficulties projecting weekly scores on graphs.** In module 1 patients are required to project their weekly scores on graphs after completing weekly ODSIS and OASIS measures. Some participants expressed concerns about forgetting the instructions for completing the graphs. To address this, the adaptation team added instructions on how to complete the graphs to the graph form itself.	No further alterations were made to the adapted material for Group 3.

Feedback from participants was used to iteratively refine the adapted protocol. The absence of any further modifications for Group 3 suggests that the refinements made based on feedback from Groups 1 and 2 were successful in improving the comprehensibility and suitability of the adapted protocol.

#### Quantitative feedback

**Fig 2:**
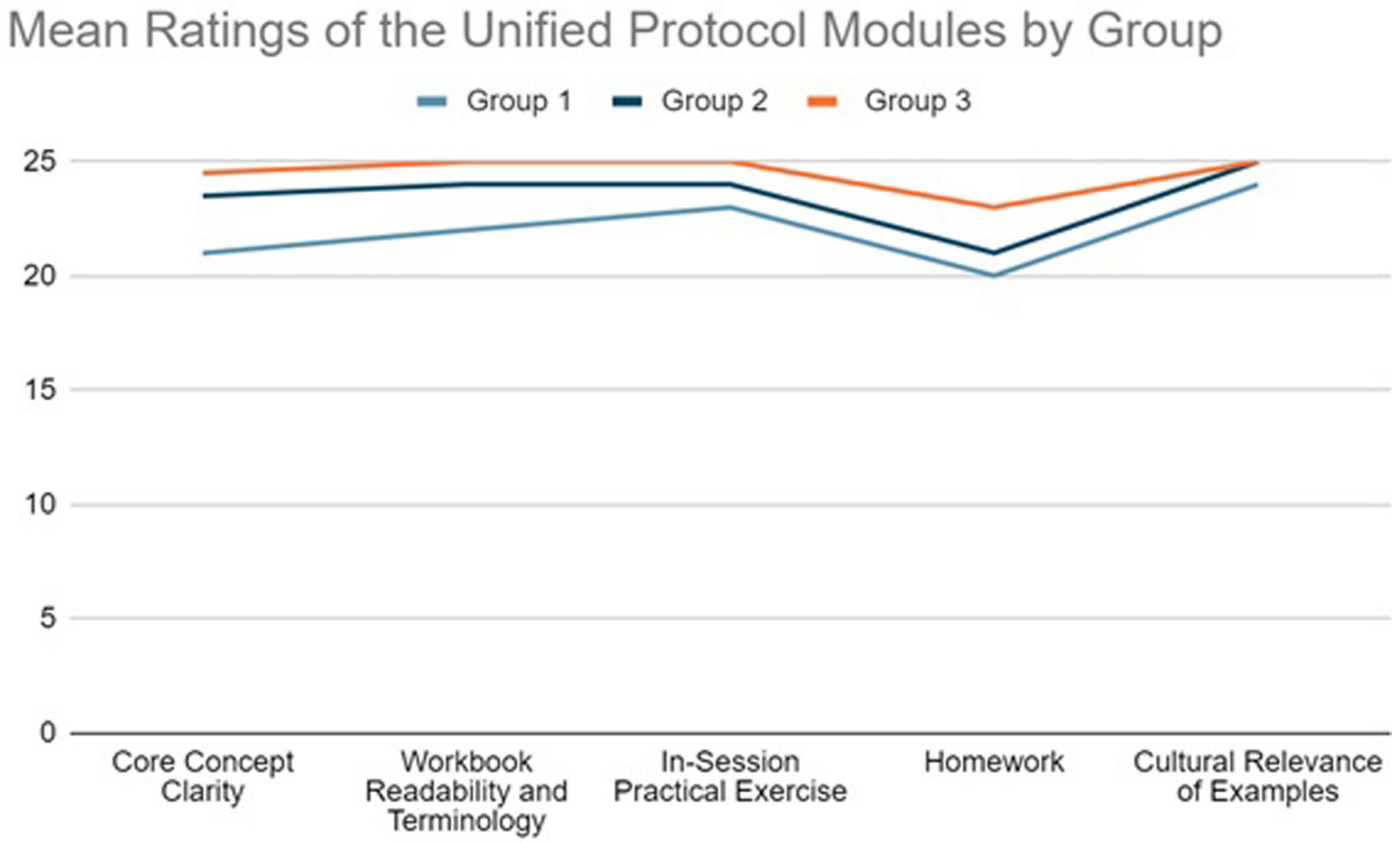
Graph 1. Mean ratings of the Unified Protocol modules by groups (n = 15).

Fig 2 shows that group 3 (n = 5) rated all aspects of the UP modules more highly than groups 1 and 2 (n = 5 each), but the differences were small. The highest mean ratings by group 3 suggests that the incorporation of the feedback appeared helpful for the participants. The quantitative results corroborate the findings of the qualitative results discussed above.

A repeated-measures ANOVA was performed to evaluate the effect of UP on depression, anxiety, difficulties in emotion regulation, and negative and positive affect at pre-, mid-, and post-treatment phases. The means and standard deviations are presented in Table 6. The results revealed statistically significantly difference between time points (BAI: F[1.43,20.05] = 8.79, η2 = 0.38, P = 0.004; BDI: F[2,28] = 8.48, η2 = 0.37, P = 0.001; WASA: F[2,28] = 74.28, η2 = 0.84, P<0.001; DERS: F[2,28] = 14.21, η2 = 0.50, P<0.001; PANAS (positive): F[1.14,16.07] = 199.72, η2 = 0.93, P<0.001; PANAS (negative): F[2,28] = 303.68, η2 = 0.95, P<0.001; OASIS: F(1, 14) = 112.596, p = .000, η² = .889; ODSIS: F(1, 14) = 23.819, p = .000, η² = .630). The result of pairwise comparisons with Bonferroni test indicated a non-significant reduction in dependent variables’ means from pre- to mid-treatment for (BAI: 4.00 P = .43; BDI: 9.40 P = .12; 17.80, P = .01; WASA: 2.00, P = 0.84); and DERS: 2.46, P = 1.00) but significant for positive PANAS (12.26, P<0.001), negative affect (16.00, p<0.001), OASIS: (9.80, P = .01) and ODSIS: 10.80, p = .000). Mean change scores showed that the treatment group achieved a significantly greater magnitude of change from pre- to post-treatment for all variables (BAI: 15.00, P = 0.003; BDI: 17.80, P = .01; WASA: 9.80, P<0.001; DERS: 40.73, P<0.001; PANAS Positive: -18.86, P<0.001; PANAS Negative: 23.80, P<0.001; OASIS: (8.26, P = .001) and ODSIS: 7.60, P = .000). To see group differences over time see the simple linear regression analysis in the S2 Appendix.

**Table 6:** Descriptive statistics for outcome variables.

Variable	Pre-treatment		Mid-treatment		Post-treatment	
	*M*	*SD*	*M*	*SD*	*M*	*SD*
BAI	36.66	10.11	32.65	15.45	21.66	9.58
BDI	38.20	15.55	28.20	5.77	20.40	10.10
DERS	120.53	29.98	118.06	13.72	79.80	21.75
WASA	18.20	1.61	16.20	2.90	8.40	1.68
PANAS (P)	16.46	1.72	28.73	2.43	35.33	3.71
PANAS (N)	40.20	3.58	24.20	2.51	16.40	2.41
OASIS	16.00	2.72	10.80	1.89	7.60	1.24
ODSIS	13.46	4.17	9.80	1.01	8.26	.88

M = Mean, SD = Standard Deviation; BAI = Beck Anxiety Inventory; BDI = Beck Depression Inventory; DERS = Difficulty in Emotion Regulation Scale; WASA = Work and Social Adjustment Scale, PANAS_P = Positive, and Negative Affect Scale-Positive; PANAS_N = Positive, and Negative Affect Scale-Negative; ODSIS = Overall Depression Severity and Impairment Scale; OASIS = Overall Anxiety Severity and Impairment Scale

## Discussion

The primary objective of this study was to describe the process of culturally adapting the UP, a transdiagnostic cognitive-behavioral intervention designed to treat a wide range of emotional disorders, within the socio-cultural milieu of Pakistan.

The adaptation of the UP to a specific sociocultural context represents a significant advancement in efforts to provide accessible and effective interventions for individuals experiencing emotional distress in resource-limited settings. The UP’s transdiagnostic focus on disruptive emotional behaviors makes it well-suited for use in a variety of cultural contexts [11]. Furthermore, the UP’s inherent flexibility allows it to be tailored to address the key intervention targets that most directly influence daily functioning in specific populations [59].

A critical stage in the cultural adaptation of the UP was the restructuring of the workbook. This involved incorporating culturally relevant graphical illustrations and examples to engage participants and improve their understanding. The content was also adapted to suit varying literacy levels, ensuring accessibility to those with lower education. Furthermore, homework sheets, which played a vital role in supplementing therapy sessions, were adjusted according to their literacy level to make them more appropriate and relevant in the local setting.

The adaptation test revealed important findings regarding the comprehensibility of the adapted-UP materials and the facilitative effect of prior discussion, a factor synonymous with effective therapeutic interventions [60]. However, challenges with homework completion, cited in other contexts, were also noted [44, 59]. Factors such as the perceived difficulty or time requirement can hinder engagement with homework tasks, while motivational deficits further impede consistent completion of assignments [61]. In the Pakistani context, cultural norms may impact how clients perceive homework. In this setting, there is often an emphasis on meeting the needs of one’s family and community [62]. This focus on the well-being of the collective could potentially result in decreased motivation to complete homework tasks that are not seen as directly benefiting the client’s social or familial connections. Moreover, societal attitudes toward mental health might cause clients to be reluctant to participate in activities such as homework that could reveal their condition to others. A strong therapeutic alliance based on trust and a culturally sensitive understanding of these dynamics becomes even more crucial for encouraging clients’ active involvement in homework assignments [63]. Therefore, despite these challenges, the adaptation team deemed it important to retain the homework component in the therapeutic process considering empirical evidence emphasizing its significance [64, 65].

While the sample size precludes formal statistical testing, a slight increase in ratings is observed in Graph 1 after the Unified Protocol (UP) refinement, suggesting potential benefits for clarity and user experience. Furthermore, the iterative nature of this study enabled the researchers to adopt a critical and reflective approach to the adaptation process. By gathering feedback from participants and using this feedback to inform modifications to the adapted protocol, the researchers were able to improve the quality and cultural appropriateness of the adapted protocol. This iterative refinement process allowed the researchers to test the adapted protocol early on, identify and address potential problems, and collect feedback from the target population to ensure that the protocol was accessible and likely to be successful.

Preliminary adaptation test findings show that UP significantly decreases anxiety and depression levels while increasing work and social functioning. These outcomes align well with UP’s therapeutic objectives and theoretical underpinnings, supporting findings from previous studies [3, 66]. The findings also suggest that UP can reduce emotional regulation difficulties. This lends credence to the application of emotional regulation strategies in managing emotions, a technique that’s especially useful for people overcoming mental health issues [67]. Furthermore, improved control over emotions can lead to reduced symptoms of both depression and anxiety [66]. Our findings mirrored observations from UP studies conducted in the USA, and low-resource settings with populations similar to ours (e.g., Iran, Colombia). Notably, these studies also reported significant fluctuations in both positive and negative effects during the mid and post-assessment phases [21, 23, 66, 68].

### Limitations

It is important to acknowledge the limitations of the present study. Although the current study identified language, literacy, and sociocultural variables that facilitated the cultural appropriateness of the intervention, further evidence can be obtained through the assessment of the protocol’s feasibility and acceptability among participants. This information is currently lacking from the adaptation testing phase. Future studies should prioritize investigating feasibility and acceptability in community hospitals to gain a more nuanced view of real-world implementation of the modified UP.

The current study’s smaller sample size allows for focused monitoring of the intervention’s adaptation process, but it limits the generalizability of findings, especially regarding participants from lower educational backgrounds. The importance of assessing the UP-treatment’s adaptability for diverse literacy levels is acknowledged. To address this concern, proactive modifications were made to the intervention materials using simpler language and incorporating visual aids. However, a more comprehensive assessment of understandability across different educational backgrounds will require future research with larger and more representative samples.

Additionally, the present study focused on evaluation of adaptations from the patient’s perspective at the testing phase. However, an important area that remains unrevealed is how mental health professionals conceptualize and explain key UP concepts and incorporate UP principles into their clinical practice. Because only one therapist provided treatment in the current study, broader research is needed to capture the perspectives of a wider range of providers and gain valuable insights into the best use of UP tailored to the local clinical contexts.

## Conclusion

The implementation of the UP in the Pakistani cultural context has the potential to significantly improve the therapeutic landscape for transdiagnostic disorders, however, successful implementation will require ongoing refinement and training to address the challenges of adapting an intervention to a new cultural context and balance theoretical integrity with cultural sensitivity. This research heralds the beginning of culturally contextualized mental health interventions for common psychological afflictions in developing countries.

## Supporting information

S1 Appendix Complete interview guide. This appendix provides the complete interview guide used at each stage of the adaptation process.
(DOCX)

S2 Appendix Supporting tables. This appendix includes two tables: Table S1: Summary of Key Findings with Representative Participant Quotations. Table S2: Simple Linear Regression to Provide Group Differences.
(DOCX)
