# Supplementary material for: Adaptation of unified protocol treatment for transdiagnostic disorders in Pakistan: A heuristic framework
Source: PLoS One. 2024 Sep 30;19(9):e0308981. doi: 10.1371/journal.pone.0308981 (PMC11441672; doi:10.1371/journal.pone.0308981)
Supplement: S1 Appendix — This appendix provides the complete interview guide used at each stage of the adaptation process. (DOCX) [file pone.0308981.s001.docx]

**Semi-structured Interview Questions**

**Pre-Adaptation Phase**

**Mental Health Professionals (N = 4)**

1. In your clinical experience here in Pakistan, what are the most prevalent psychological disorders you encounter among your patients?
2. Cognitive Behavioral Therapy (CBT) is a widely used treatment approach. How have you observed CBT impacting various mental health disorders compared to other treatment methods in your practice?
3. Pakistan's cultural background is unique. In your opinion, how does this cultural context influence how mental health disorders manifest in your patients?
4. To better serve your patients' needs, what treatment modifications have you found most necessary when dealing with mental health disorders in Pakistan?

## **Community professionals (N = 2)**

1. Can you describe how people in our community generally view mental health?
2. There can be a lot of shame or secrecy surrounding mental health concerns. How do you see this playing out in terms of people seeking treatment for mental health disorders here?
3. Pakistan has a unique cultural context. Can you elaborate on how mental health treatments are currently delivered and adapted to fit the needs of our community?
4. Diagnosing and treating mental health disorders accurately can be complex. What are some of the biggest challenges faced in this area here in Pakistan?

5. Is there anything else you'd like to share about mental health and its treatment in our community?

**Patients (N = 3)**

1. How do you define your illness or condition in your own words?
2. Does your illness have any symbolic meaning to you? For example, are there cultural or personal associations you make with your condition?
3. Have you ever received any psychological treatment in the past, such as therapy or counselling?
4. If yes, what specific methods or approaches were used in your past treatments?
5. How effective were these past treatments in helping you manage your condition?
6. In what ways did past treatments impact your daily life, both positively and negatively?
7. Considering your experiences, what aspects of past treatments would you have liked to see done differently?
8. What kind of treatment approach would you prefer moving forward?
9. How do you envision culturally appropriate treatment being delivered? What would that look like to you?

**Prior to adaptation all stakeholders were asked to assess the protocol keeping this question in mind:**

Considering the cultural aspects of mental healthcare in Pakistan, what recommendations would you make for adapting it to be more effective here?

**Stage 2 - Preliminary Adaptation Design**

**Quantitative Assessment (Local Sources)**

**Comprehension:**

- To what extent do the instructions clearly explain how to complete the worksheets? (1 = Not at all clear, 5 = Very clear)
- How easy is it to understand the language used in the workbook? (1 = Very difficult, 5 = Very easy)
- Are the cultural references in the workbook easy to understand? (1 = Not at all easy, 5 = Very easy)

**Cultural Appropriateness:**

- How well do the images and examples reflect the target culture? (1 = Not at all well, 5 = Very well)
- Are there any references or content that could be offensive or confusing to the target culture? (1 = Yes, a lot, 5 = No, none)
- Is the overall tone of the workbook appropriate for the target audience? (1 = Not at all appropriate, 5 = Very appropriate)

**Overall Usefulness:**

- How helpful is this workbook in understanding the treatment program? (1 = Not helpful at all, 5 = Very helpful)
- How likely are you to recommend this workbook to others in the target population? (1 = Not likely at all, 5 = Very likely)
- How confident are you that this workbook will be useful for the target population? (1 = Not confident at all, 5 = Very confident)

**Semi-Structured Interview Questions**

**Stage 3 - Preliminary Adaptation Testing (N = 15)**

- How was your overall experience reading about different psychological concepts and techniques in Urdu?
- Did you find it easy or difficult to understand the material?
- In the content you read, what concepts were difficult to understand? Were there any concepts you grasped easily?
- Did you encounter any new vocabulary or ideas that you weren't familiar with before reading the materials?
- How easy were the sentences to understand?
- Did you find the sentence structure overly complex?
- How did you find the overall workbook in terms of its usability and helpfulness?
- Did you feel overwhelmed by the amount of material?
- Did you find the workbook enjoyable to work through?
- Did the examples provided in the workbook connect with your personal experiences or general observations?
- Did you find the examples to be culturally appropriate?
- Can you recall a specific example that stood out to you as particularly helpful?
- How did you feel about the quizzes at the end of each chapter?
- Describe your experience completing the weekly homework assignments.
- What challenges did you face in understanding the content of the treatment program?
- What aspects of the program were difficult for you to grasp?
- How can these challenges be overcome to improve the learning experience?

**Quantitative Assessment
Rate the module on the following statements from 1 to 5 (1 = Strongly Disagree, 5 = Strongly Agree)**

1. To what extent were the core concepts in the UP Workbook easy to understand?
2. How easy was it to read and understand the language used in the UP Workbook?
3. How helpful were the in-session practical exercises in the UP Workbook for understanding the concepts?
4. How effective were the homework exercises in the UP Workbook in helping you apply the concepts to your daily life?
5. How well did the examples used in the UP Workbook reflect your own cultural background and experiences?
