# Supplementary material for: Adaptation of unified protocol treatment for transdiagnostic disorders in Pakistan: A heuristic framework
Source: PLoS One. 2024 Sep 30;19(9):e0308981. doi: 10.1371/journal.pone.0308981 (PMC11441672; doi:10.1371/journal.pone.0308981)
Supplement: S2 Appendix — This appendix includes two tables: Table S1: Summary of Key Findings with Representative Participant Quotations. Table S2: Simple Linear Regression to Provide Group Differences. (DOCX) [file pone.0308981.s002.docx]

**Appendix**

**Table 1: Themes, Subthemes and Example Quotes from Participant Feedback on Adapted UP**

| **Themes** | **Subthemes** | **Description** | **Example Quotes** |
| --- | --- | --- | --- |
| **Comprehensibility of the UP** | Content | Participants found explanations clear, concise, and used everyday language. | "For the most part, I found the material to be quite manageable." |
|  | Sentence Structure | Participants found the writing style engaging and easy to follow, with clear and concise sentences. | "The sentences were straightforward and not overly complicated." |
| **Facilitative effect of prior discussion** | Enhanced Comprehension | Discussions helped clarify doubts and pre-expose participants to key terms, leading to better understanding. | "Talking about the content before reading it improves my comprehension. There were a few terms I didn't know, but by discussing them beforehand, it became easier to understand when they appeared in the reading." |
|  | Enhanced Information Retention | Discussions led to a more active learning experience, fostering deeper understanding and memory consolidation. | "The discussions before reading really enhanced my understanding of the material. It was not just passive reading; we were actively discussing the concepts, which made them feel more tangible and memorable." |
| **Cultural relevance of the UP** | Relatable Examples | Participants found examples relatable to their own experiences and cultural background. | "The examples in the workbook were excellent. They didn't feel like generic situations; they felt like something that could really occur within our culture. It made it much simpler to grasp the concepts." |
|  | Culturally Appropriate | Sensitivity to cultural context enhanced the learning experience. | "I felt that the materials' examples truly took into account our cultural heritage. It wasn't as if they were attempting to enforce foreign ideas on us. It felt pertinent and respectful." |
| **Assessment and Practice: Quizzes and Homework** | Effectiveness of Quizzes | Participants found chapter quizzes helpful for reinforcing learning and assessing comprehension. | "I found the end-of-chapter quizzes to be very beneficial. They prompted me to review the material I had just covered, serving as a helpful tool for evaluating my understanding." |
|  |  |  |  |
|  | Effectiveness of Homework Assignments | Participants found homework assignments valuable for applying concepts to real-world scenarios. | "The weekly homework assignments offered an excellent opportunity to apply what I had been learning. They allowed me to see how the concepts could be utilized in real-life situations." |
| **Challenges with Homework Completion** | Time Constraints | Busy schedules made it difficult to take out time for the homework. | Busy schedules made it difficult for some participants to dedicate time for homework. |
|  | Motivation | Lack of immediate results could hinder motivation for some participants. | "At times, I struggle with being motivated to finish the homework assignments, especially if I don't notice immediate changes. It's difficult to keep focused on long-term advantages.” |

**Table 2 -** Regression results using time and group as the criteria (N = 15)

| **Effect** | **Time 2** | | | | | **Time 3** | | | | | **Group 2** | | | | | **Group 3** | | | | |
| --- | --- | --- | --- | --- | --- | --- | --- | --- | --- | --- | --- | --- | --- | --- | --- | --- | --- | --- | --- | --- |
|  | β | SE | 95% CI | | *p* | β | SE | 95% CI | | *p* | β | SE | 95% CI | | *p* | β | SE | 95% CI | | *p* |
|  |  |  | LL | UL |  |  |  | LL | UL |  |  |  | LL | UL |  |  |  | LL | UL |  |
| **BDI** | -.38 | 3.47 | -17.48 | -3.44 | .004 | -0.81 | 3.47 | -29.42 | -15.38 | .000 | .21 | 3.40 | -.94 | 12.81 | .08 | .20 | 3.40 | -1.28 | 12.48 | .10 |
| **BAI** | -.22 | 3.59 | -14.93 | -.40 | .039 | -.70 | 3.59 | -25.59 | -11.06 | .000 | .22 | 3.59 | -1.33 | 13.19 | .10 | /13 | 3.59 | -3.79 | 10.73 | .34 |
| **ODSIS** | -.67 | .48 | -5.57 | -3.62 | .000 | -1.04 | .48 | -8.04 | -6.09 | .000 | .69 | .48 | -.64 | 1.30 | .49 | 1.24 | .48 | -.37 | 1.57 | .22 |
| **OASIS** | .78 | -.54 | -6.19 | -3.00 | .000 | .78 | -.97 | -9.86 | -6.67 | .000 | .13 | .78 | -.46 | 2.72 | .15 | .00 | .78 | -1.59 | 1.59 | 1.00 |
| **WASA** | -.26 | .77 | -4.22 | -1.10 | .001 | 1.01 | .77 | -11.89 | -8.77 | .000 | -.05 | .77 | -2.16 | .96 | .44 | -.02 | .77 | -1.76 | 1.36 | .79 |
| **DERS** | -.11 | 5.75 | -16.95 | 6.28 | .359 | .73 | 5.75 | -45.15 | -21.91 | .000 | .23 | 5.75 | -1.02 | 22.22 | .07 | .26 | 5.75 | .51 | 23.75 | .04 |
| **PANAS-P** | .65 | .99 | 9.92 | 13.94 | .000 | 1.09 | .99 | 17.85 | 21.94 | .000 | -.06 | .99 | -3.14 | .87 | .26 | -.06 | .99 | -2.67 | 1.34 | .50 |
| **PANAS-N** | -.68 | 1.60 | -17.84 | -11.35 | .000 | 1.02 | 1.60 | -25.10 | -18.62 | .000 | .10 | 1.60 | -2.64 | 3.84 | .71 | .10 | 1.60 | -1.10 | 5.37 | .19 |

*Note:* BDI = Beck Depression Inventory, BAI = Beck Anxiety Inventory, ODSIS = Overall Depression Severity and Impairment Scale, OASIS = Overall Anxiety Severity and Impairment Scale, WASA = Work and Social Adjustment Scale, DERS = Difficulties in Emotion Regulation Scale, PANAS-P = Positive and Negative Affect Scale - Positive, PANAS-N = Positive and Negative Affect Scale - Negative. Total N = 15. CI = confidence interval; LL = lower limit; UL = upper limit. **p* < .05, ***p* < .01, ****p* < .001

A simple linear regression analysis was conducted to examine the relationship between time (baseline, mid-, and post-assessment) on all the study variables. For BAI and BDI scores the model was significant F (4, 40) = 7.247, p < .000), F (4, 40) = 11.792, p < .000), explaining 42.0% and 54.1% of the variance in BAI and BDI scores (R² = .420, .541). This indicates that the scores were significantly lower at both mid- and post-assessment compared to baseline, indicating an improvement in symptoms and behaviors over time. Specifically, BAI scores decreased by an average of 7.667 points at mid- and 18.333 points at post-assessment, BDI scores decreased by 10.467 points at mid-time and 22.400 points at post-assessment. Likewise, for ODSIS and OASIS, the model was significant F (4, 40) = 55.553, p < .000), F (4, 40) = 28.309, p < .000) explaining 84.7% and 73.9% of the variance in ODSIS and OASIS scores (R² = .847, .739), respectively. ODSIS scores decreased by 4.600 points at mid and 7.067 points at post-assessment, OASIS scores decreased by 4.600 points at mid and 8.267 points at post-assessment suggesting that time has a significant negative impact on ODSIS and OASIS scores. The model was significant for WASA F (4, 40) = 48.369, p < .000), explaining 82.9% of the variance in WASA scores (R² = .829). WASA scores decreased by 2.667 points at mid and 10.333 points at post-assessment. The DERS scores indicate that the model was statistically significant F (4, 40) = 11.142, p < .000), explaining 52.7% of the variance in DERS scores (R² = .527), and the scores decreased by 5.333 points at mid and 33.533 points at post-assessment. To examine the relationship between time and PANAS positive the model was again significant F (4, 40) = 101.365, p < .000), explaining 91.0% of the variance in positive PANAS scores (R² = .910). The model was significant for PANAS negative F (4, 40) = 48.684, p < .000), explaining 83.0% of the variance in negative PANAS scores (R² = .830). Positive PANAS scores significantly increased by 11.933 points at midpoint and 19.867 points at post-test, indicating that positive emotions increased over time as opposed to negative PANAS scores decreasing significantly by 14.600 points at midpoint and 21.867 points at post-test. These data suggest that time plays an important role in predicting outcomes for all variables tested, but treatment groups remained insignificant, indicating that there was no significant difference between them.
